# Conference Reports: SIXTH INTERNATIONAL CONFERENCE ON HIGH TEMPERATURES-CHEMISTRY OF INORGANIC MATERIALS, Gaithersburg, MD, April 3–7, 1989

**DOI:** 10.6028/jres.095.032

**Published:** 1990

**Authors:** J. W. Hastie

**Affiliations:** Metallurgy Division, Materials Science and Engineering Laboratory, National Institute of Standards and Technology, Gaithersburg, MD 20899

This conference was the sixth of a series, sponsored by the International Union of Pure and Applied Chemistry (IUPAC) Commission II.3 on High Temperature and Solid State Chemistry, and which is held about every 3 years.

The NIST meeting represented only the second occasion that this conference series had been held in the U.S.A. Attendance, exceeding 170, included participants from 19 countries, and 130 papers were presented.

## 1. About the Conference

The conference program emphasized the basic chemical science and measurement issues underlying the characterization, processing, and performance of materials at high temperatures. Each of the major classes of materials was considered, including high performance alloys, ceramics, composites, and specialized forms such as films, coatings, clusters, powders, slags, fluxes, etc. in addition, individual substances, namely the elements and their compounds, were discussed in detail. Seven plenary lectures and 68 invited talks were given as well as 61 poster presentations and computer-based demonstrations. Also, Prof. Leo Brewer, one of the foremost pioneers of the field, gave an overview of the conference proceedings together with his perspective on the "Role of Chemistry in High-Temperature Materials Science and Technology." During the conference sessions, many of the hot issues of the day were also discussed, including cold fusion, high-temperature superconductors, low pressure production of diamond films, etc.

Participation by the leading international researchers in the field was particularly strong in the materials-related areas of measurement techniques, thermochemistry and models, processing and synthesis, and performance under extreme environments. Of special interest were the topics on databases and phase equilibria models, processing—mainly from the vapor phase, and high power laser-materials interactions.

The conferees were welcomed by Dr. Lyle Schwartz, Director of the Institute for Materials Science and Engineering (IMSE) (now Materials Science and Engineering Laboratory), who also gave an overview of pertinent NIST and IMSE research activities. Prof. Jean Drowart of the Free University of Brussels, Belgium, addressed the meeting on behalf of IUPAC and gave a fascinating account of "7000 Years of High Temperature Materials Chemistry."

A few representative technical highlights from each of the main conference sessions are given in the following discussion.

## 2. Advances in Measurement Techniques

**Figure 1:**
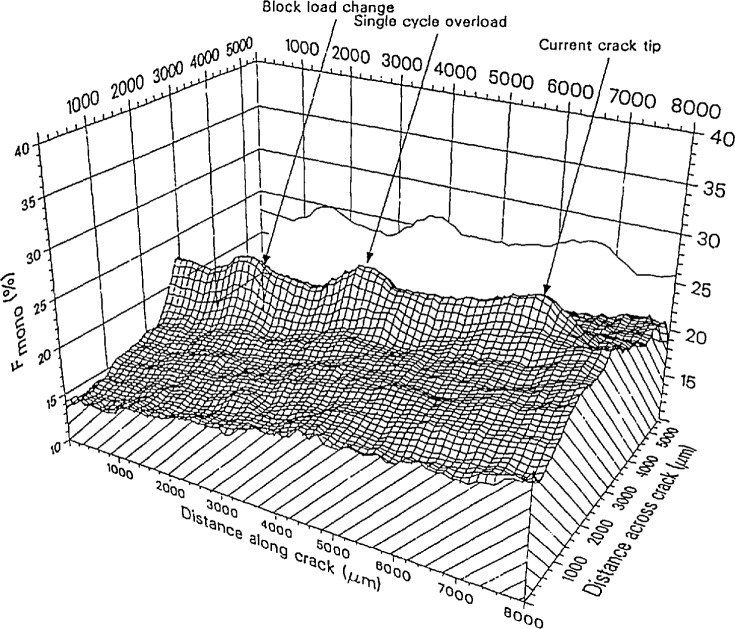
A map of the monoclinic phase fraction of a zirconia specimen subjected to an apphed stress and crack growth. The stress history of the material is revealed in the extent and degree of transformation of the transformed zone. Large stresses induce a larger transformed zone around the crack tip that remains after the crack tip moves forward. (Taken from Rosenblatt et al., paper 4.)

**Figure 2:**
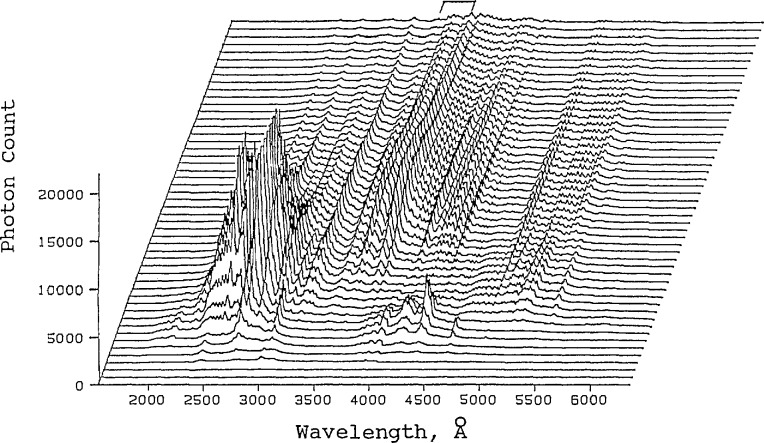
Time-resolved emission spectra from a laser produced plasma plume generated off a specialty steel alloy target. Each trace represents a 20 ns exposure spectrum covering the spectral range of 1850 to 6200 A. Each successive trace is delayed by 20 ns and the 50 traces shown cover the first 1 *μ*s of the plume. The laser energy is 3.38 J and the ambient gas is argon at 0.015 Torr at room temperature. (Taken from Kim, paper 5.)

Three areas were given special emphasis. These were spectroscopic probes, diffractometry, and physicochemical methods. The types of spectroscopic probes discussed included Raman and related laser spectroscopic methods for *in situ* molecular-level or phase-specific monitoring of hot surfaces. Examples were considered in the areas of corrosion, oxide superconductor processing, and in Raman imaging of ceramic crack suppression due to phase transformation toughening (see fig. 1). An interesting novel application of *in situ* optical emission spectroscopic analysis of molten steel, using a laser-induced plasma-forming technique, was also discussed (see fig. 2). These effectively nonintnisive methods also have potential as process monitoring probes for intelligent processing in addition to their utility in experimental systems.

In the area of diffractometry, *in situ* analysis of material structures at high temperatures, using x-ray and neutron sources, was described. Atom probe chemical analysis on alloy surfaces using field-ion microscopy was also discussed.

Physicochemical techniques have traditionally been key to the characterization of materials at high temperatures and significant recent advances have occurred in this area. Methods have been developed which effectively eliminate containment problems. For instance, with liquid metals, transient microsecond time scale techniques have been applied to accurate measurements of melting points and heat capacities at very high temperatures. For steady state measurements, electromagnetic levitation may be used as, for instance, with emissivity and optical constant measurements. Another transient technique that was discussed by a number of researchers throughout the conference is the pulsed laser-heating approach to the production of vapor species for mass and optical spectroscopic characterization.

## 3. Thermochemistry and Models

**Figure 3:**
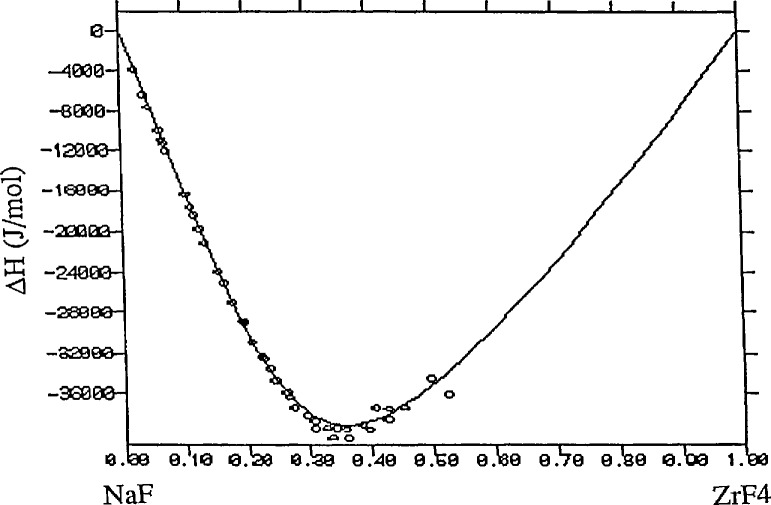
Enthalpy of mixing of the NaF-ZrF_4_ system. Data points are experimental and line is calculated using an associated liquid model. (Taken from Gaune-Escard et al., paper 35.)

**Figure 4:**
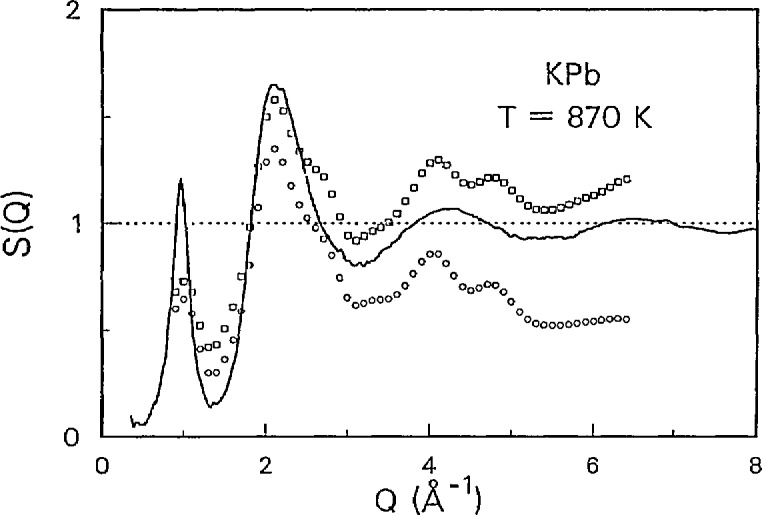
Structure factors, *S*(*Q*), for liquid BLPb. Solid line: *S*(*Q*) from diffraction measurements on SEPD; Points *S*_Δ_(Q)=_−Δ_∫*S*(*Q,E*) from inelastic scattering measurements on LRMECS: (□)Δ=40 meV, (o)Δ=5 meV. (Taken from Saboungi et al., paper 27.)

This session was particularly well represented by the leaders in the field. Progress on development of thermodynamic databases was reviewed by researchers from the United States, U.S.S.R., Canada, France, Sweden and the United Kingdom. While the databases developed thus far are incomplete they are still sufficiently extensive to allow their use in thermochemical and phase equilibria models for many high-temperature alloy, ceramic, composite, slag, glass, and other systems. A key element in these models is the description of non-ideal mixing, present in many practical systems. Among the various models considered, those accounting for ordering or formation of Uquid associates appear particularly promising (see fig. 3). In one of the presentations, direct experimental (neutron diffraction) evidence was presented for ordering in Uqud alloys (see fig. 4). Many papers were presented dealing with experimental determinations of thermochemical data and applications of the data to materials process development.

## 4. Processing and Synthesis

**Figure 5:**
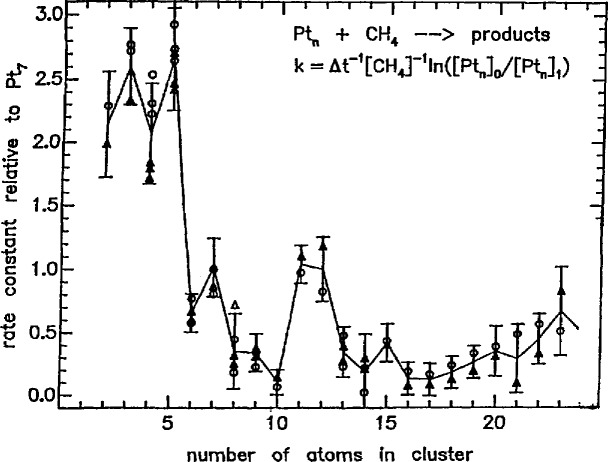
Reaction rate of Pt*_x_* with CH_4_, normalized to Pt_τ_. (Taken from Kaldor et al., paper 77.)

The chemical basis for high temperature processing and synthesis of materials is a rapidly growing area of research and representative work in the field was discussed. An area of significant promise for the design of new or improved materials is that of molecular/atomic clusters. These species, with properties intermediate between molecular and bulk material, are key reaction intermediates to most deposition and condensation processes. They also serve as model structures for surfaces owing to their intrinsic high ratio of surface to bulk atoms. Their unique reactivity as a function of cluster size was indicated by several speakers (see fig. 5).

**Figure 6:**
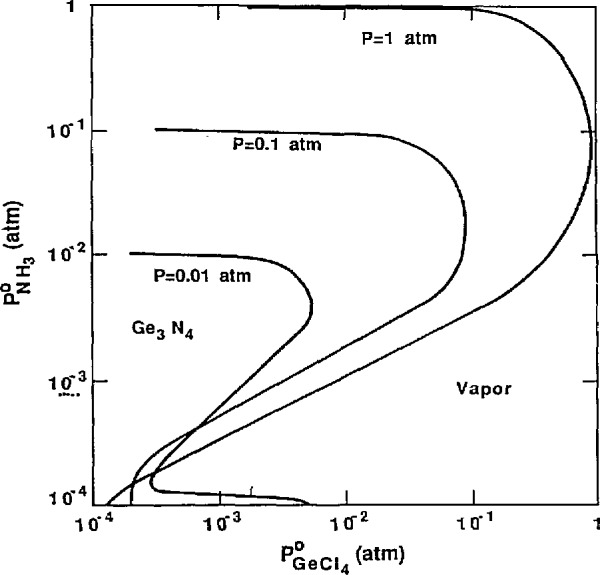
Phase fields for deposition of Ge_3_N_4_ as a function of deposition temperature and the feed ratio $P^{0}{}_{{\text{NH}}_{3}}/P^{0}{}_{\text{GeCl}4}$, for the GeCl_4_-NH_3_-N_2_ system. *P*=1 atm and $P^{0}{}_{\text{GeCl}4}=10^{-2}$ atm. (Taken from Anderson et al., paper 105.)

The session on CVD and other vapor phase-based processes was particularly exciting. Thermochemical, kinetic, transport models, whereby the processing of films (diamond, semiconductor, ceramic, alloy, etc.) could be optimized, were described (see fig. 6).

## 5. Performance Under Extreme Environments

**Figure 7:**
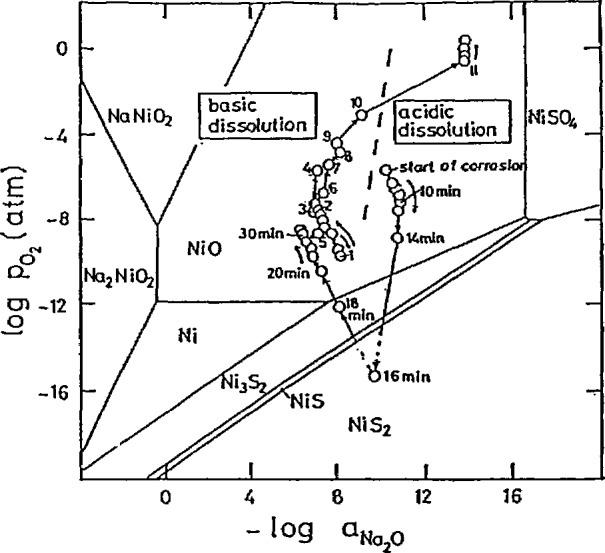
Trace of basicity and oxygen activity measured for preoxidized 99% Ni covered with a Na_2_SO_4_ film at 900 °C in 0.1% SO_2_-O_2_ gas atmosphere (preoxidized at 900 °C for 4 h in O_2_). Numbers designate reaction time in hours except as indicated. Severe corrosion conditions. (Taken from Rapp, paper 115.)

The important related areas of hot and high temperature corrosion were discussed for both alloy and ceramic materials. In particular, the key role of chemical reaction and solubility was demonstrated (see fig. 7).

**Figure 8:**
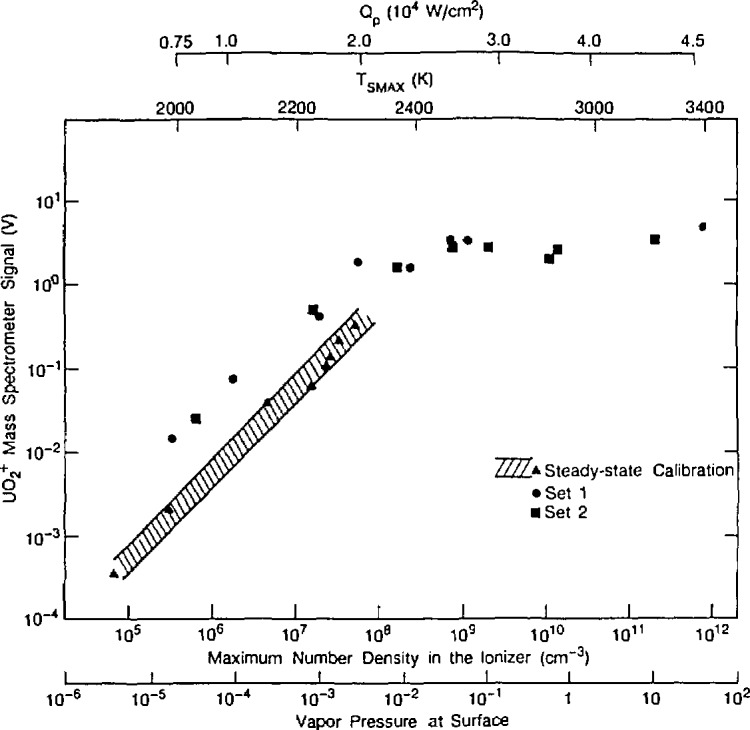
Maximum UO_2_^+^ signals from the mass spectrometer for laser pulses of varying strength. *Q*_p_ is the peak absorbed power density, and *T*_smax_ is the measured maximum surface temperature in the pulse. The scale designating the maximum number density in the ionizer of the mass spectrometer was calculated from measured ion intensities and the vapor pressure (Torr) is that of UO_2_ at the peak surface temperature. The hatched area represents the range of results of the steady-state calibrations, (Taken from Olander, paper 123.)

Another area where materials are subject to extreme conditions is that of laser-materials interactions. There are many areas of science and technology that require an improved understanding of this interaction, including design of laser resistant materials, laser deposition of films, laser etching for electronic devices, laser stimulated chemical processing, laser welding, and laser heating for containerless studies of thermochemistry at ultra-high temperatures. This latter case has special significance to providing thermodynamic data for nuclear reactor excursions (see fig. 8) and for materials data for advanced aerospace applications.

## 6. Additional Information

A three volume proceedings (1350 pages) is being published by Humana Press, Clifton, NJ. Many of the conference presentations will appear in these volumes. Also included are a few articles, not presented at the conference, in order to provide a more complete coverage of certain topics. This will be the first generally available publication for this subject area and the proceedings should be of considerable interest to researchers, students, and others interested in the scientifically challenging, and technologically indispensable, interplay between materials and high temperatures.

The next meeting in the series is scheduled to be held in 1991 in Orleans, France and will be chaired by J. P. Coutures.

## 7. List of Papers Presented at the Conference

### ADVANCES IN MEASUREMENT TECHNIQUES

#### Spectroscopic Probes

1. *R. J. M. Anderson* and J. C. Hamilton—(Sandia National Lab., United States) Nonlinear Optical Spectroscopy as a Probe of Properties and Processes at Surfaces and Interfaces
2. *K. F. McCarty*, D. R. Boebme, D. S. Ginley, E. L. Venturini, and B. Morosin (Sandia National Labs., United States) High-Temperature Processing of Oxide Superconductors: A Raman Scattering Study
3. *M. D. Allendorf—* (Sandia National Labs., United States) Temperature Measurements in Silica-Laden Flames by Spontaneous Raman Scattering
4. *G. M. Rosenblatt* and D. K. Veirs —(Lawrence Berkeley Lab., United States) Recent Developments nsing Imaging Detectors for Raman Characterization of High Temperature Materials
5. *Y. W.* Am—(Lehigh Univ., United States) Laser Plasma Plume Analysis in High Temperature Condensed Phases
6. *Y. Shiraishi* and K. Kusabiraki—(Tohoku Univ., Japan) Infrared Spectrum of High Temperature Melts by Means of Emission Spectroscopy
7. *I. R. Seattle*, N. Binsted, W. Levason, J. S. Ogden, M. D. Spicer, and N. A. Young—(Univ. Southhampton, United Kingdom) EXAFS, Matrix Isolation and High Temperature Chemistry

#### Diffractometry

8. *H. F. Franzen* and S.-J. Kim—(Iowa State Univ., United States) High Temperature X-Ray Diffraction and Landau Theory Investigations of Thermal Symmetry-Breaking Transitions: The W Point of Fm3m and the Structure of NbN_1−_*_x_*
9. *R. D. Shull* and J. P. Cline—(NIST, United States) High Temperature X-Ray Diffractometry of Ti-Al Alloy Phase Transitions
10. *J. Faber, Jr.* and R. L. Hitterman—(Argonne National Lab., United States) High Temperature insitu Neutron Diffraction Studies of the Defect Structure of Non-stoichiometric Oxides
11. *R P. Camus—*(NiST, United States) Field-Ion Microscopy and Atom Probe Chemical Analysis

#### Pbyslco-Chenucal Methods

12. *A. Cezairliyan—*(NIST, United States) A Microsecond-Resolution Transient Technique for Thermophysical Measurements on Liquid Refractory Metals
13. *R. H. Hauge*, S. Krishnan, G. P. Hansen, and J. L. Margrave—(Rice Univ., United States) Emissivities and Optical Constants of Electromagnetically Levitated Liquid Metals as Functions of Temperature and Wavelength
14. *M. Shamsuddin*^1^—(Banaras Hindu Univ., India) Techniques for Measurement of Thermodynamic Properties of Chalcogenides
15. *M. V. Korvbov*^1^, E. B. Rudnyi, O. M. Vovk, E. A. Kibicheva and L. N. Sidorov—(Moscow State Univ., U.S.S.R) Ion Equilibria—A New Technique for Measurement of Low O_2_ Partial Pressures
16. *M A. Frisch* and E. A. Giess —(IBM Yorktown Heights, United States) Kinetics of Water Desorption from Glass Powders Studied by Knudsen Effusion Mass Spectrometry
17. *K. A. Gingerich*, M. J. Stickney, and M. S. Chandrasekharaiah—(Texas A&M Univ., United States) A Novel Vapor Source for the Thermodynamic Study of Alloys with a High Temperature Mass Spectrometer
18. *D. Bostrom, B. Lindbtom*, E. Rosen, and M. Sodelund —(Univ. Umea, Sweden) The Zero Point Technique: An Improved Method to Determine Equilibrium Oxygen Partial Pressure of Slow Reacting Chemical Systems at High Temperatures
19. *K. Zmbov*, J. W. Hastie, D. W. Bonnell, and D. L. Hildenbrand—(Boris Kidric Inst., Yugoslavia) Mass Spectrometric Analysis of LiF and AgCl Vaporization and Temperature Dependent Electron Impact Fragmentation

### THERMOCHEMISTRY AND MODELS

#### Databases and Phase Equilibria Models

20. *L. V. Gurvich—* (Institute of High Temperature, U.S.S.R.) Reference Books and Databanks on the Thermodynamic Properties of Inorganic Substances
21. *M. W. Chase* and R. D. Levin—(NIST, United States) Thermodynamic Properties of the Alkaline Earth Hydroxides: A JANAF Case History
22. *I. Ansara—*(Domaine Univ., France) Thermodynamic Modeling of Solution Phases and Phase Diagram Calculations
23. *A. D. Pelton*, W. T. Thompson, and C. W. Bale—(Ecole Polytechnique, Canada) Thermodynamic Databases for Multicomponent Solution-Modeling and Data Evaluations
24. *M. H. Rand*, R. H. Davies, A. T. Dinsdale, T. G. Chart, and T. I. Berry—(Harwell Lab. Didcot, United Kingdom) Application of MTDATA to the Modeling of Multicomponent Equilihria
25. *B. Jonsson* and B. Sundman —(Royal Institute of Technology, Sweden) Thermochemical Applications of THERMO-CALC
26. *M. Seapan* and J. Y. Lo—(Oklahoma State Univ., United States) A Simulation Model to Predict Slag Composition in a Coal Fired Boiler
27. *M. L. Saboungi*, G. K. Johnson, and D. L. Price—(Argonne National Lab., United States) Ordering in Some Liquid Alloys
28. *M. Ramanaihan*, S. Ness, and D. Kalmanovitcb—(Univ. of North Dakota, United States) New Techniques for Thermochemical Phase Equilibrium Predictions in Coal Ash Systems
29. *R. G. Reddy* and H. Hu—(Univ. Nevada-Reno, United States) Modeling of Viscosities of Alkali-, Alkaline-Earth Metal Oxide and Silicate Melts
30. *M. W. Chase*, F. Glasser, and A. Bernstein—(NIST, United States) PC Demonstration of Thermodynamic Databases
31. *L. V. Gurvich*, V. S. Iorish and V. S. Youngman—(Institute of High Temperature, U.S.S.R.) Extended and Updated Data Bank on Thermodynamic Properties of Inorganic Substances
32. *D. W. Bonnell* and J. W. Hastie—(NIST, United States) A Predictive Slag Phase Equilibria Model
33. *H. M. Ondik—*(NiST, United States) The NIST—ACerS Ceramic Phase Diagram Data Base
34. *M. Gaune-Escard*, J. P. Bros, and G. Hatem—(Univ. de Provence, France) Thermosalt, A Thermodynamic Data Bank for Moitcn Mixtures
35. *M. Gaune-Escard* and G. Hatem—(Univ. de Provence, France) Thermodynamic Modelling of High Temperature Melts and Phase Diagram Calculations

#### Phase Equilibria Experimental and Applications

36. *P. W. Gilles* and G. F. Kessinger—(Univ. of Kansas, United States) The High Temperature Vaporization and Thermodynamics of the Magncli Phases of the Titanium-Oxygen System
37. *C. B. Alcock—*(Univ. Notre Dame, United States) Strontium Oxide Activities in Oxide Ceramics
38. *J.-C. Lin* and Y. A. Chang—(Univ. Wisconsin, United States) Thermodynamics, Kinetics and Interface Morphology of Reactions Between Metals and III-V Compound Semiconductors
39. *E. Kaldis*—(ETH-Zurich, Switzerland) Thermodynamic Instabilities In High-Temperature Compounds With Intermediate Valence
40. C. K. Mathews^1^—(Indira Gandhi Centre for Atomic Research, India) Recent Studies on Thermochemistry and Phase Equilihria in Alkali Metal Systems
41. *M. Iwase*, M. F. Jiang, and E. ichise—(Kyoto Univ., Japan) Thermochemistry of the System MO+MX_2_+Fe_x_O (M=Ca, Sr, Ba, and X=F, Cl)
42. A. i. Saitzev, N. V. Korolev, and B. M. Mogutnov^1^—(I. P. Bard in Research Institute, U.S.S.R) Thermodynamic Properties and Phase Equilihria at High Temperatures in CaO-CaF_2_, Al_2_O_3_-CaO, and CaF_2_-Al_2_O_3_-CaO Systems
43. *H. Ipser*, R. Krachler, G. Hanninger, and K. L. Komarek—(Univ. of Vienna, Austria) Thermodynamic Properties of NiAs-Type Co_1±_*_x_*Sb and Ni_1_*_±_*_x_Sb
44. A. I. Saitzev, M. A. Semchenko, and B. M. Mogutnov^1^—(I. P. Bardin Central Research Institute, U.S.S.R.) Thermodynamic Properties and Phase Equilibria at High Temperatures in Fe-Cr and Fe-Mn Systems
45. *M. Pelino*, A. Florindi, and M. Petroni—(Univ. dell’Aquila, Italy) Study of the Decomposition Process of a-Goethite by Thermal Gravimetry “in Vacuo”
46. *L. P. Cook*, E. R. Plante, D. W. Bonnell, and J. W. Hastie—(NIST, United States) Reaction of Liquid LI, Al and Mg with Gaseous Cl_2_, O_2_ and F_2_
47. *R. H. Hauge*, M. Sampson, J. L. Margrave, J. Porter, and G. Reynolds—(Rice Univ., United States) Mass Spectrometric Studies of the Vaporization Behavior of SrZrO_3_, SrHfO_3_, Yttria Stabilized Hafnia and Ir_0.4_Al_0.6_
48. *K. Hilpert* and M. Miller - (Nuclear Research Center, Federal Republic of Germany) Chemical Vapor Transport and Compiexation in the NaI-ScI_3_ System
49. *K. Hilpert*, S. R. Dharwadkar, D. Kobertz, V. Venugopal, and H. Nickel—(Nuclear Research Center, Federal Republic of Germany) Differential Thermal Analysis and Knudsen Effusion Mass Spectrometry in the Determination of Phase Equilibrium Diagrams in Nickel Based Superalloys
50. *J. C. Liu*, M. P. Brady, and E. D. Verink, Jr.—(Univ. of Florida, United States) Phase Stability and Kinetics Study in High Temperature Oxidation of Nb Ti-Al Alloys
51. *D. Hoelzer* and F. Ebrahimi—(Univ. of Florida, United States) Phase Stability in the Nh-Ti-Al Ternary System
52. *E. M. Foltyn*—(Los Alamos National Lab., United States) Allotropie Transitions in Neptunium Metal hy Differential Thermal Analysis
53. *B. M. Mogutnov*^1^ A. I. Saitzev, and N. V. Korolev—(I. P. Bardin Central Research Institute for Ferrous Metallurgy, U.S.S.R.) The Vapor Pressures and the Heats of Sublimation of CaF_2_ and SrF_2_
54. *B. M. Mogutov*^1^ and A. I. Saitzev—(I. P. Bardin Central Research Institute for Ferrous Metallurgy, U.S.S.R.) The Vapor Pressures and the Heats of Sublimation of Some Rare Earth Metals
55. *J. M. Leitnaker*, R. W. Nichols, and B. S.. Lankford—(Martin Marietta Energy Systems Oak Ridge, United States) Reactions of Aluminum with Uranium Fluorides and Oxyfhiorides

#### Basic Data Determinations

56. *J. Drowart*, A. V. Gucht, S. Smoes—(Free Univ. Brussels, Belgium) Mass Spectrometric Investigation of Systems Far From Thermodynamic Eqnilthrium Using the Knudsen Effusion Method
57. *L. N. Gorokhov*, A. M. Emelyanov, and M. V. Milushin—(High Temp. Inst.,U.S.S,R.) Knudsen Effusion Mass Spectrometry Determination of Metal Hydroxide Stabilities
58. *V. L. Stolyarova—*(Silicate Inst. Academy of Sciences, U.S.S.R.) Mass Spectrometric Study and Calculation of Thermodynamic Properties of Glass-Forming Oxide Systems
59. *C. E. Myers*, G. A. Murray, R. J. Kematick, and M. A. Frisch—(State Univ. New York at Binghamton, United States) Comparison of Knudsen Vaporization by Magnetic and Quadrupole Mass Spectrometric Techniques
60. *J. G Edwardsaiid* J. K. R Weber—(Univ. of Toledo, United States) Vaporization Chemistry in the CaS-Ga_2_S_3_ System
61. *G. Balducci*, G. De Maria, G. Gigli, and M. Guido—(Univ. di Roma ‘La Sapienza’, Italy) Vaporization Behavior of Molten Alkali Metal Metavanadates
62. *J. K. Gibson* and R. G. Haire—(Oak Ridge National Lab., United States) Knudsen Effusion Investigation of the Thermal Decomposition of Transplutonium Hydrides
63. *P. W. Gilles* and M. A. Williamson—(Univ. of Kansas, United States) Vaporization Chemistry of the Vanadium Selenides
64. *R. G. Haire* and J. K. Gibson—(Oak Ridge National Lab., United States) On the Enthalpies of Sublimation of Einsteinium and Fermium
65. *D. L. Hildenbrand*, K. H. Lau, and R. D. Brittain—(SRI International, United States) Mechanistic Aspects of Metal Sulfate Decomposition Processes
66. *K. Hilpert* and K. Ruthardt—(Nuclear Research Centre, Federal Republic of Germany) Determination of the Enthalpy of Dissociation of the Molecule CrPb by High Sensitivity Knudsen Effusion Mass Spectrometry
67. *P. D. Kleinschmidt* and K. Axler—(Los Alamos National Lab., United States) Activity and Free Energy of Formation of the Compound CaCsCl_3_
68. *P. C. Nardine*, R. A. Schiffman, and J. K. R. Weber—(Intersonics, Inc., United States) Vapor Pressure of Boron
69. *G. N. Papatheodarou* and L. Nalhandian—(Institute of Chemical Engineering and High Temperature Chemical Processes, Greece) Raman Spectra and Vibrational Analysis of the Fe_2_Cl_3_, FeAlCl_6_, au_2_cl_6_ and AuAlCl_6_ Vapor Molecules
70. M. Shamsuddin and A. Nasar^1^—(Banaras Hindu Univ., India) Thermodynamic Properties of Cadmium Telluride
71. *V. L. Stalyarova*, I. Y. Archakov, and M. M. Shultz—(Institute of Silicate Chemistry of the Academy of Sciences, U.S.S.R.) High Temperature Mass Spectrometric Study of the Thermodynamic Properties of Borosilicate Systems
72. *M. E. Jacox* and W. E. Thompson—(NIST, United States) The Production and Spectroscopy of Small Polyatomic Molecular Ions Isolated in Solid Neon
73. M. Shamsuddin,^1^ A. Nasar, and V. B. Tare—(Banaras Hindu Univ., India) Electrical Conductivity and Defect Structure of Cadmium Telluride
74. *M. Gaune-Escard* and A. Bogacz—(Univ. de Provence, France) Calorimetric Investigation of NdCl_3_ and of NdCl_3_-MCl Mixtures
75. *J. P. Bros*, D. El Allant, M. Gaune-Escard, and E. Hayer—(Univ. de Provence, France) Enthalpies of Formations of Ni- and Pd-Based Ternary Alloys
76. *C. B. Coughanowr*, T. J. Anderson, and J. J. Egan—(Univ. of Florida, United States) Thermodynamic Investigation of the Al-Sb and Al-In Systems by Solid State Electrochemistry

### PROCESSING AND SYNTHESIS

#### Clusters as Reaction Intermediates and Model Structures

77. *A. Kaldar*—(Exxon Research and Engineering, United States) Clusters as Intermediates for New Materials
78. *F. W. Froben*, T. M. Chandrasekhar and J. Kolenda—(Freie Univ. Berlin, Federal Republic of Germany) Ouster Production by Laser Material Interaction With Optical Spectroscopic Characterization
79. *M. Vala*, T. M. Chandrasekhar, and J. Szczepanski—(Univ. of Florida, United States) Spectroscopy and Structure of Small Carbon Clusters
80. *K. G. Weil* and A. Hartman—(Technische Hochschule Darmstadt, Federal Republic of Germany) Mechanism of Cluster Formation during Evaporation of Alloys
81. *K. Hilpert* and D. Kath—(Nuclear Research Centre, Federal Republic of Germany) Investigation of Small Alkali Metal Clusters by Knudsen Effusion Mass Spectrometry using Broad Band Photoionization
82. *T. C. DeVare* and J. L. Gole—(James Madison Univ., United States) Oxidation of Small Metal Clusters
83. *R. S. Berry*, H.-P. Cheng, and J. Rose—(Univ. of Chicago, United States) Freezing and Melting of Metallic and Salt-Like Ousters
84. *E. Blaisten-Barojas* and M. Nyden—(NIST. United States) Thermal Fragmentation of Long Carbon Chains
85. *K. A. Gingerich*, J. E. Kingcade, Jr., and I. Shim—(Texas A&M Univ., United States) Bond Energies and Nature of Bonding in Small Transition Metal Semiconductor Clusters
86. *P. J. Ficalaro* and J. H. Hawley—(Rensselaer Polytechnic Institute, United States) Heterogeneous Formation of Aluminum Vapor Clusters

#### Nuclcation and Growth of Small Particles

87. *J. Schoonman*, R. A. Bauer, and J. G. M. Becht—(Delft Univ. Technology, The Netherlands) Laser-Chemical Vapor Precipitation of Ultrafine Ceramic Powders: Si and Si_3_N_4_
88. *N. Shima* and K. Yoshihara—(Idemitsu Kosan Central Research Labs., Japan) Laser Production of Metallic Fine Particles from Organometallic Compounds
89. *J. L. Katz* and M. D. Donohue—(Johns Hopkins Univ., United States) Nucleation with Simultaneous Chemical Reaction
90. *P. R. Buerki*, T. Troxler, and S. Leutwyler—(Univ. Bern, Federal Republic of Germany) Synthesis of Ultrafine Si_3_N_4_ Particles by CO_2_-Laser Induced Gas Phase Reactions
91. *M. R. Zachariah* and H. G. Semerjian—(NIST, United States) Experimental and Numerical Studies of Refractory Particle Formation in Flames: Application to Silica Growth

#### Processing, Mainly from the Vapor Phase

92. *K. E. Spear—*(Pennsylvania State Univ., United States) The Role of High Temperature Chemistry in CVD Processing
93. *C. Bernard*—(ENSEEG Domaine Univ., France) Thermochemical Modeling of Vapor Deposition
94. *Y. K. Raa* and Y. Do—(Univ. of Washington, United States) Modeling of Chemical Vapor Deposition (or Etching) in Closed Systems
95. *F. W. Smith*, M. Sommer, and K. Mui—(City College of the City Univ. of New York, United States) Thermodynamic Analysis of the Chemical Vapor Deposition of Diamond Films
96. *J. E. Butler—*(Naval Research Lah., United States) The Chemical Vapor Deposition of Synthetic Diamond
97. *E. Schnedler* and H. Greiner—(Phillips GmhH Forschungslaboratorium Aachen, Federal Republic of Germany) Modelling of High Temperature Transport Reactions
98. *J.-O. Carlssan*—(Uppsala Univ., Sweden) Area Selective and Phase-Selective CVD on Patterned Substrates
99. *R. Naslain* and F. Langlais—(Lab. des Composites Thermostmcturaux, France) Fundamental and Practical Aspects of the Chemical Vapor Infiltration of Porous Substrates
100. *T. H. Baum* and C. E. Larson—(IBM Almaden Research Center, United States) Laser Chemical Vapor Deposition of High Purity Metals
101. *U. B. Pal* and S. C. Singhal—(Westinghouse R&D Center, United States) Growth of Perovskitc Films by Electrochemical Vapor Deposition
102. *J. S. Harwitz* and M. C. Lin—(U.S. Naval Research Lah., United States) Laser and Mass Spectrometric Studies of the Mechanism of Silicon Single Crystal Etching Reactions
103. *Z. A. Munir—*(Univ. of California Davis, United States) The Utilization of Combustion Processes for the Synthesis of High Temperature Materials
104. *K. L. Kamarek* and H. Blaha—(Institute of Inorganic Chemistry, Austria) The Reduction or Silica With Graphite
105. *T. J. Anderson*, J. L. Ponthenier, and F. Defoort—(Univ. of Florida, United States) Thermodynamic Analysis of Ge_3_N_4_ Chemical Vapor Deposition
106. *Z. J. Kafafi* and R. S. Pong—(Naval Research Lah., United States) The Activation of the C-H Bond of Allene by Ground State Atomic Iron
107. *T. C. DeVare*, M. L. Smith, and J. C. Fagerli—(James Madison Univ., United States) Chemical Vapor Transported Species Resulting from the Oxidation of Hot W, Mo Filaments by N_2_O, O_2_, POCI_3_, and K_2_ClO_4_

#### Process Models and Materials by Design

108. *P. J. Spencer* and H. Holleck—(Lehrstuhl fur Theoretische Hüttenkunde, Federal Republic of Germany) Application of a Thermochemical Data Bank System to the Calculation of Metastable Phase Formation During PVD of Carbide, Nitride and Boride Coatings
109. *P. R. Strutt* and G.-M. Chow—(Univ. of Connecticut, United States) Ultrafine Composite Synthesis by Laser-Indnced Reactive Evaporation and Rapid Condensation
110. *J. W. Mitchell* and G. Cadet—(AT&T Bell Labs., United States) Microwave Discharge Synthesis and Characterization of Materials
111. *J. D. Corbett*, E. Garcia, Y.-U. Kwon, and A. Guloy—(Iowa State Univ., United States) Chemical Clusters from Solid State Systems at High-Temperatures—Interstitials as a Means to Stability and Versatility
112. *P. K. Khowash* and D. E. Ellis—(Northwestern Univ., United States) Impurity Defect Structure in Alpha-Alumina
113. *N. Zacchetti*, G. Fierro, G. M. Ingo, A. Mazzarano, S. Sturlese—(Centre Sviluppo Material! SpA, Italy) High Temperature Stability of CCO_2_-Y_2_O_3_ Stabilized Zirconia Plasma Spray Powders: XPS and DTA Investigations
114. *N. Zacchetti* and G. M. Ingo—(Centra Sviluppo Materiali SpA, Italy) XPS Investigation on the Chemical Structure and Growth Model of Amorphous Silicon Nitride (a-SiN*x*)

### PERFORMANCE UNDER EXTREME ENVIRONMENTS

#### Hot Corrosion

115. *R. A. Rapp—*(Ohio State Univ., United States) Hot Corrosion of Materials
116. *R. L. Janes*—(Naval Research Lab., United States) Oxide Acid-Base Reactions in Ceramic Corrosion
117. *N. S. Jacobson*, J. E. Marra, E. R. Kreidler, and M. J. McNallan,—(NASA Lewis Research Center, United States), High Temperature Reactions of Ceramics and Metals with Chlorine and Oxygen
118. *N. Birks*, D. L. Rishel, and F. S. Pettit—(Univ. of Pittsburg, United States) Erosion and Corrosion of Metals in Sulfurous Atmospheres
119. *I. Tomizuka*, H. Numata, H. Harada, Y. Koizumi, and M. Yamazaki—(National Research Institute for Metals, Japan) Effects in Processing History and Minor Element Contents on Hot-Corrosion Behavior of a Power-Metallurgically Prepared Nickel-Base Superalloy
120. *W. Boersma-Klein* and J. Kistemaker—(FOM-Institute for Atomic and Molecular Physics, The Netherlands) Material Transport at the Interface of a Graphite Wall and a U-C-F Gas/Liquid Mixture
121. *E. Franconi*, M. Rubel, and B. Emmoth—(Associazione EURATOM-ENEA sulfa Fusione Centra Ricerche Energia Frascati, Italy) Deuterium Implanted in C+SiC and CL5890PT Materials
122. *V. U. Kodash*, P. S. Kisley, and V. J. Shemet—(Institute of Superhard Materials, Academy of Sciences, U.S.S.R.) High Temperature Oxidation of Molybdenum Aluminosilicides

#### High Power Laser-Materials Interactions

123. *D. R. Olander*, S. K. Yagnik, and C. H. Tsai—(Univ. of California, United States) Laser-Pulse-Vaporization of Uranium Dioxide and Other Refractory Materials
124. *J. L. Lyman*, D. A. Cremers, R. D. Dixon, R. C. Estler, G. K. Lewis, R. E. Muenchausen, N. S. Nogar, M. Piltch—(Los Alamos National Lab., United States) Direct Laser/Materials Interaction: Laser Ablation of Superconductor Materials and Laser Welding
125. *M. J. Berry*, T. D. Kunz, R. F. Menefec, and L. G. Fredin—(Rice Univ., United States) Laser Probe Absorption Spectroscopy Measurements on Laser Induced Plumes
126. *Y. Nishina* and A. Kasuya—(Materials Research Institute, Japan) Space/Time Resolved Spectroscopic Analysis on High Power Laser-Materials Interaction
127. *R. W. Dreyfus—*(IBM, Yorktown Heights, United States) Interactive Effects in Exclmer Laser Pbotoablation
128. *K.-S. Lyu*, J. Kralik, and Y. W. Kim—(Lehigh Univ., United States) Laser Produced High Temperature States for Iron
129. *P. K. Schenck*, D. W. Bonneli, and J. W. Hastie—(NIST, United States) Insitu Analysis of Laser-Induced Vapor Plumes

### CONFERENCE WRAP-UP

130. *L. Brewer*—(Univ. of California Berkeley, United States) A Conference Overview with a Personal Perspective on the Role of Chemistry in High Temperature Materials Science and Technology

## Footnotes

1 Paper presented in absentia.

